# Allosteric Modulation of Muscarinic Acetylcholine Receptors

**DOI:** 10.2174/157015907781695946

**Published:** 2007-09

**Authors:** Karen J Gregory, Patrick M Sexton, Arthur Christopoulos

**Affiliations:** Drug Discovery Biology Laboratory, Department of Pharmacology, Monash University, Clayton, Victoria, 3800, Australia

**Keywords:** Acetylcholine, allosteric interaction, G protein-coupled receptor, molecular modeling, muscarinic acetylcholine receptor, mutagenesis, radioligand binding, structure-activity studies, ternary complex model.

## Abstract

Muscarinic acetylcholine receptors (mAChRs) are prototypical Family A G protein coupled-receptors. The five mAChR subtypes are widespread throughout the periphery and the central nervous system and, accordingly, are widely involved in a variety of both physiological and pathophysiological processes. There currently remains an unmet need for better therapeutic agents that can selectively target a given mAChR subtype to the relative exclusion of others. The main reason for the lack of such selective mAChR ligands is the high sequence homology within the acetylcholine-binding site (orthosteric site) across all mAChRs. However, the mAChRs possess at least one, and likely two, extracellular allosteric binding sites that can recognize small molecule allosteric modulators to regulate the binding and function of orthosteric ligands. Extensive studies of prototypical mAChR modulators, such as gallamine and alcuronium, have provided strong pharmacological evidence, and associated structure-activity relationships (SAR), for a “common” allosteric site on all five mAChRs. These studies are also supported by mutagenesis experiments implicating the second extracellular loop and the interface between the third extracellular loop and the top of transmembrane domain 7 as contributing to the common allosteric site. Other studies are also delineating the pharmacology of a second allosteric site, recognized by compounds such as staurosporine. In addition, allosteric agonists, such as McN-A-343, AC-42 and *N*-desmethylclozapine, have also been identified. Current challenges to the field include the ability to effectively detect and validate allosteric mechanisms, and to quantify allosteric effects on binding affinity and signaling efficacy to inform allosteric modulator SAR.

## INTRODUCTION

G protein-coupled receptors (GPCRs) account for 1 - 3% of the human genome, are abundantly expressed throughout the central nervous system (CNS) and periphery, and represent the major targets for approximately 30% of all medicines on the world market. However, current CNS-based GPCR drug discovery has a higher than average attrition rate with respect to translating fundamental research to the clinic [41]; this is likely due to two reasons, namely, an insufficient mechanistic understanding of the complexities of CNS GPCR-mediated signaling and a lack of selective pharmacological tools for targeting therapeutically relevant GPCRs. As a consequence there are many GPCR-based drug discovery programs aiming to develop more selective compounds, both as tools to probe GPCR biology and also as potential therapeutic leads. The traditional approach to GPCR-based drug discovery has been to focus on targeting that region of the receptor utilized by the receptor’s endogenous ligand, i.e., the “orthosteric” site [80]. However, it is now recognized that GPCRs possess topographically distinct, allosteric binding sites, and that ligands that bind to these sites (allosteric modulators) offer tremendous potential for more selective and/or effective therapies than conventional orthosteric ligands. This brief review will focus on one of the best-studied families of GPCRs with respect to the phenomenon of allosteric modulation, namely, the muscarinic acetylcholine receptors.

## MUSCARINIC ACETYLCHOLINE RECEPTORS (mAChRs): A BRIEF OVERVIEW

The mAChRs belong to the Family A (rhodopsin-like) subclass of GPCRs. Pharmacological and genetic studies have identified five distinct mAChR subtypes, classed M_1_-M_5_. The M_1_, M_3_ and M_5_ subtypes preferentially couple to the G_q/11 _family of G proteins, resulting in phospholipase C activation, hydrolysis of inositol phosphates and mobilization of intracellular Ca^++^ stores. In contrast, the M_2_ and M_4_ subtypes preferentially couple to the pertussis toxin-sensitive G_i/o _family of G proteins, resulting in the inhibition of adenylyl cyclase and subsequent cAMP formation. Although these generalizations speak to the best-characterized signaling pathways associated with the mAChRs, they should by no means be taken as absolutes. All five mAChR subtypes are known to couple pro-miscuously to multiple G proteins, usually in a cell background dependent manner, and have been linked to additional intracellular pathways, including activation of mitogen activated protein kinases, Rho GTPases, nitric oxide synthases, multiple phospholipases, and the modulation of a variety of potassium, calcium and chloride ion channels [58].

The mAChRs are widely distributed throughout the periphery and the CNS. Activation of peripheral mAChRs leads to increases in exocrine secretion, contraction of cardiac and smooth muscle (gastrointestinal tract and lungs), and reduced heart rate. Within the CNS, a far more complex array of physiological behaviors is thought to be mediated by the mAChRs, depending on their distribution and localization [13]. M_1_ mAChRs are predominantly expressed post-synaptically in forebrain regions including the cerebral cortex, hippocampus and striatum [68, 69, 76, 80, 88]. These receptors have long been associated with cognitive deficits linked to neurodegenerative disorders, such as Alzheimer’s disease, and as such selective agonists of the M_1_ mAChR have been pursued as a potential avenue for treatment of dementia-related conditions [32]. The M_2_ mAChR is located pre-synaptically on both cholinergic and non-cholinergic neurons [30, 88] in the brainstem, hypothalamus/thalamus, hippocampus, striatum and cortex [68, 69, 80], and generally serves an inhibitory function on the release of neurotransmitters. It has been suggested that enhancing synaptic ACh levels by selectively inhibiting M_2_ autoreceptors may be beneficial in the treatment of psychosis and Alzheimer’s disease, and an attractive alternative to the currently used cholinesterase inhibitors for the latter disorder [20]. M_3_ mAChRs are expressed at relatively low levels in a number of regions including the cortex, striatum, hippocampus, hypothalamus/thalamus. These receptors have been particularly associated with appetite regulation, and the M_3_ receptor is currently a potential target for treatment of obesity and other metabolic disorders [7, 34, 69, 109]. M_4_ mAChRs are predominantly found presynaptically in the striatum, hippocampus, cortex and hypothalamus/thalamus [9, 69, 80]. There is the potential that M_4_ mAChR selective antagonists may control tremor associated with Parkinson’s disease, whilst agonists may be developed as analgesics, due to the regulation of neurotransmitter release in both cholinergic and non-cholinergic neurons [23, 113], and as novel antipsychotics, due to regulation of the dopaminergic system [1, 91]. Finally, M_5_ mAChRs are discretely expressed at low levels in the brain, in particular in the ventral tegmental area [103, 110] as well as co-localised with D_2_ dopamine receptors in the substantia nigra pars compacta [107]. They are also implicated in the control of vasodilatation of cerebral blood vessels [108]. M_5_ mAChRs are associated with slow activation of dopaminergic neurons and subsequent reward behaviors [111], and as such M_5_ selective agents may be used to treat addiction and psychosis, as well as maintain cerebral blood flow in the certain pathophysiological states such as cerebral ischemia.

The pharmacological characterization of mAChRs is not a straightforward task due to the high level of sequence conservation within the orthosteric binding site across all five mAChR subtypes. As a consequence, there are very few orthosteric agonists and antagonists that exhibit high selectivity for one subtype to the relative exclusion of others. The traditional approach to pharmacological delineation of which mAChR governs a given response has thus been to use a combination of compounds, generally antagonists, to build up a receptor profile. For example, the M_1_ mAChR is generally defined as having high affinity for pirenzepine and 4-DAMP (4-diphenylacetoxy-N-methyl-piperidine methiodide), whilst having low affinity for methoctramine and himbacine. M_2_ mAChRs have high affinity for methoctramine, himbacine and AF-DX 384 (5,11-dihydro-11-[2-[2-[(N,N-dipropylaminomethyl)piperidin-1-yl]ethyl-amino]-carbonyl] 6H-pyrido[2,3-b][1,4]benzodiazepin-6-one) and have low affinity for pirenzepine and 4-DAMP. A high affinity for 4-DAMP, and low affinity for pirenzipine, methoctramine and himbacine suggests the involvement of the M_3_ mAChR. The presence of the M_4_ mAChR can be determined using PD102807 and the toxin, MT3. The M_5_ mAChR has been notoriously difficult to identify pharmacologically, however both AF-DX 384 and AQRA741 (11-((4-[4-(diethylamino)butyl]-1-piperidinyl)acetyl)-5,11-dihydro-6H-pyrido(2,3-b)(1,4)benzodiazepine-6-one) have the lowest affinity (at least 10 fold lower) for this subtype than any other.

Given the high degree of sequence homology within the mAChR orthosteric site, and the current paucity of suitably selective mAChR orthosteric ligands, it stands to reason that alternative approaches are required to better achieve target specificity. All five mAChRs possess at least one [25], and likely two [62], extracellular allosteric binding sites for small molecules, and significant efforts have been underway, especially within the last decade and a half, in trying to understand the nature of these sites. The most important challenge in this field remains the ability to detect and quantify the myriad of possible allosteric effects that can arise when two ligands occupy a receptor at the same time.

## DESCRIBING ALLOSTERIC INTERACTIONS

The binding of an allosteric ligand to its site will change the conformation of the receptor, which means that the “geography” of the orthosteric site and any other potential receptor-ligand/protein interfaces, can also change. As a consequence, the binding affinity and/or signaling efficacy of the orthosteric ligand is likely to be modulated, either in a positive or negative manner. The simplest allosteric GPCR model assumes that the binding of an allosteric ligand to its site modulates only the affinity of the orthosteric ligand; this model is referred to as the allosteric ternary complex model (ATCM; Fig. (**1A**)). Within the framework of an ATCM, the interaction is governed by the concentration of each ligand, the equilibrium dissociation constants (K_A_ and K_B_, respectively) of the orthosteric and allosteric ligands, and the “cooperativity factor” α, which is a measure of the magnitude and direction of the allosteric interaction between the two conformationally linked sites [24, 94]. A value of α <  1 (but greater than 0) indicates negative cooperativity, such that the binding of an allosteric ligand inhibits the binding of the orthosteric ligand. Values of α > 1 indicate positive cooperativity, such that the allosteric modulator promotes the binding of orthosteric ligand, whereas values of α = 1 indicate neutral cooperativity, i.e. no net change in binding affinity at equilibrium. Because the two sites are conformationally linked, the allosteric interaction is reciprocal, i.e., the orthosteric ligand will modulate the binding of the allosteric ligand in the same manner and to the same extent.

Since the simple ATCM describes the effect of the modulator only in terms of changes in orthosteric ligand affinity, and vice versa, the stimulus that is generated by the ARB ternary complex is assumed to be no different to that imparted by the binary AR complex. In general, many mAChR modulators studied to date appear to behave in a manner consistent with this simple ATCM. However, there is no *a priori* reason why the conformational change engendered by an allosteric modulator in the GPCR does not perturb signaling efficacy in addition to, or independently of, any effects on orthosteric ligand binding affinity. Indeed, changes in the predominance of drug screening methods from a focus on (orthosteric) radioligand binding to functional assays has unmasked modulators whose actions cannot be sufficiently described by the simple ATCM; it is clear that these latter compounds can affect the signaling capacity of orthosteric agonists [75]. Moreover, there are allosteric ligands that not only modulate orthosteric ligand signaling, but also act as agonists in their own right [54]. To account for such allosteric effects on efficacy, the ATCM has been extended into an allosteric “two-state” model (ATSM; Fig. (**1B**)) [38]. This model describes GPCR function in terms of: a) the ability of the receptor to constitutively isomerize between active (R*) and inactive (R) states, as determined by the isomerization constant, L; b) the ability of orthosteric *and* allosteric ligands to modify this transition between states, i.e., to act as either agonists or inverse agonists, which is governed by the parameters α and β; c) the ability of each ligand to allosterically modulate the binding affinity of the other, governed by the “binding cooperativity” parameter, γ; d) the ability of either ligand to modulate the transition to an active receptor state when both ligands are bound, governed by the “activation cooperativity” parameter, δ. While it is widely accepted that GPCRs can adopt multiple active and inactive conformations beyond the simple R and R^*^ paradigm [102], the ATSM nonetheless provides the simplest mechanistic framework with which to describe the wide array of allosteric modulator effects on receptor binding and functional properties.

These considerations suggest that allosteric modulators can be further subdivided on the basis of their phenotypic behaviors, namely, *allosteric enhancers* (of affinity, efficacy or both), *allosteric antagonists* (affinity, efficacy or both) and *allosteric agonists*. It should also be noted that there is no reason why a modulator could not express more than one of these properties concomitantly, e.g., agonism (positive or inverse) together with enhancement or inhibition of orthosteric ligand binding/function [75, 90]. Currently, it remains to be determined whether a single phenotype (modulator only) or a combination of both modulator and agonist properties is the optimal approach to treating GPCR-based diseases with allosteric ligands. Most likely, different therapies will benefit differently from one type of phenotype relative to another. Irrespective of phenotype, the most obvious advantage of allosteric ligands is the potential for greater receptor subtype selectivity, as allosteric sites need not have evolved to accommodate an endogenous ligand [17]. An additional advantage of allosteric modulators that have no agonistic activity in the absence of orthosteric ligand is the ability to retain the spatial and temporal aspects of normal (physiological) receptor function; the modulator would only exert an effect when and where the endogenous neurotransmitter or hormone is present. Furthermore, modulators with limited cooperativity will have an in-built “ceiling” level to their effect, suggesting that they may be potentially safer than orthosteric ligands if administered in very large doses.

## DETECTING ALLOSTERIC INTERACTIONS

By and large, cell-based functional assays have surpassed radioligand binding assays as primary screens for allosteric GPCR modulators. However, there are advantages and disadvantages to both types of assays when measuring allosteric modulator effects, and ideally a combination of binding and functional experiments should be used where possible. When assessing experimental data for possible evidence of allosteric effects, the following approaches are generally utilized:

### i) Assessment of the Translocation of Orthosteric Ligand Concentration-Response or Binding Curves

Simple competition between two orthosteric ligands for a common binding site predicts a strict relationship between the apparent potency of one ligand in the absence relative to the presence of the other. This relationship is defined by the factor 1+[B]/K_B_, where [B] is the antagonist concentration, and K_B_ its equilibrium dissociation constant [2, 33]. In functional assays this change in agonist potency is manifested as a progressive dextral displacement of the orthosteric agonist concentration-response curve; in binding assays this is evidenced by a complete inhibition of orthosteric radioligand binding by increasing concentrations of competitor, irrespective of the concentration of the radiolabeled probe. In contrast, because of the cooperativity that characterizes an allosteric interaction, the changes in orthosteric ligand potency in the presence of a modulator can deviate dramatically from this expectation.

**Fig. (2):**
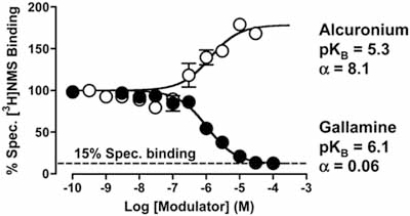
Interaction between the allosteric modulators gallamine or alcuronium with the orthosteric radioligand, [^3^H]N-methylscopolamine ([^3^H]NMS) in membranes from CHO cell stably expressing the human M_3_ mAChR. Curves superimposed on the data represent the best fit of the simple ATCM. The dashed line denotes the residual level of specific [^3^H]NMS binding in the presence of saturating gallamine concentrations.

In studies of mAChRs, it is common to see the use of the high affinity (non-selective) radiolabeled orthosteric antagonists, [^3^H]-*N*-methylscopolamine ([^3^H]-NMS) and [^3^H]-quinuclidinyl benzilate ([^3^H]-QNB), as probes of the mAChR orthosteric site. Fig. (**2**) shows the interaction between the allosteric modulators gallamine or alcuronium against the binding of [^3^H]NMS at M_2_ mAChRs. In each instance, the allosteric interaction is evidenced by the deviation of the [^3^H]NMS binding isotherm from the expectations of simple orthosteric competition. In the case of alcuronium, the specific binding of [^3^H]NMS is *increased* to due to a stabilization by the modulator of an orthosteric ligand-receptor complex characterized by a higher affinity of the radioligand for the receptor than in the absence of modulator. In the case of gallamine, specific [^3^H]NMS binding is reduced, but not completely; residual [^3^H]NMS binding is still detectable, indicating that the radioligand is able to occupy the receptor in the presence of gallamine, albeit with significantly reduced affinity. In addition to detecting allosteric ligands that modulate orthosteric ligand affinity, these types of equilibrium binding assays can also be used to quantify the allosteric effect in terms of the simple ATCM, thus providing estimates of modulator K_B_ and α (Fig. **2**). It should be noted, however, that for allosteric inhibitors with very high negative cooperativity (α approaches zero), the interaction may not be readily discernible from simple competition due to the profound reduction of radioligand affinity that ensues. In some cases, the allosteric nature of the interaction can be revealed by repeating the experiment in the presence of very high radioligand concentrations [ 57], but practical considerations may often preclude this approach.

**Fig. (3):**
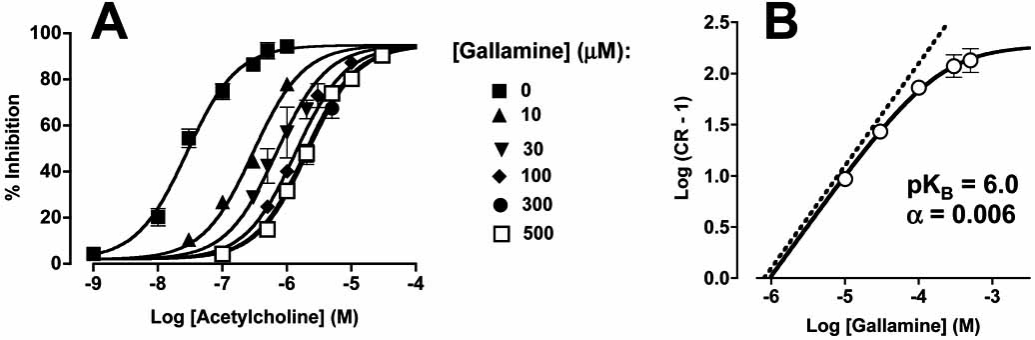
**A)** Interaction between acetylcholine and gallamine at native M_2_ mAChRs in the guinea pig electrically-driven left atrium. Data taken from [ 16]. **B)** Concentration-ratios (CR) were derived from the data in panel A and plotted in the form of a Schild regression. Solid curve denotes the fit of the ATCM to the data. Dashed line denotes the expected Schild regression for a simple competitive interaction.

Similar considerations apply to the measurement of allosteric modulator effects in functional assays. If the modulator behaves according to the simple ATCM, then the only effect that should be observed is a parallel translocation of the agonist concentration-response curve either to the left (allosteric enhancement) or the right (allosteric antagonism), with no significant change in the basal or maximum responses (but see below). In addition, if the cooperativity is limited, then the tell-tale sign of an allosteric interaction would be that the agonist curve translocation will approach a limit above which no further shifts occur, irrespective of additional increments in modulator concentration. This is illustrated in Fig. (**3A**), where the prototypical allosteric modulator, gallamine, displays a progressive inability to antagonize the effects of ACh on the guinea pig electrically-driven left atrium as the modulator concentration is increased. Often, these types of data are expressed in the form of a Schild regression [2], in which case the allosteric effect is seen as a curvilinear regression (Fig. **3B**) that asymptotes towards a value of –Logα [75]. As with binding assays, highly negative cooperative interactions may be difficult to distinguish from competitive interactions because the Schild regression will remain linear over a very large range of antagonist concentrations.

### ii) Assessment of the Maximum Attainable Response to an Orthosteric Agonist

The increased use of functional screening assays has certainly expanded the spectrum of possible allosteric effects that can be observed, specifically, by facilitating the detection of compounds that alter orthosteric ligand efficacy, as well as allosteric compounds that modify receptor activity in their own right. The most common method of detecting an allosteric modulator that affects orthosteric ligand efficacy is to monitor effects on the maximal agonist response in the presence of increasing modulator concentrations. In contrast to changes in curve translocation (agonist potency), which can reflect effects on both agonist affinity and efficacy, changes in maximal agonist responsiveness are more unambiguously attributed to modulation of agonist efficacy. Fig. (**4**) shows the interaction between the allosteric modulator, alcuronium, and the partial orthosteric agonist, pilocarpine, at human M_2_ mAChRs measured using a Cytosensor microphysiometer (which quantifies changes in whole cell extracellular acidification rates upon activation). Although the modulator is an allosteric enhancer of [^3^H]NMS binding affinity (Fig. **2**), it is clear that, when tested against pilocarpine, the same compound is an allosteric inhibitor of orthosteric agonist efficacy [112]. This is an example of the “probe-dependence” of allosteric interactions, namely, that the manifestation of cooperativity between the orthosteric and allosteric sites is totally dependent on the chemical nature of the compounds occupying the sites; the same allosteric modulator can be negatively cooperative with one orthosteric ligand, and positively cooperative with another.

In practice, the ability to optimally discern an allosteric effect on agonist efficacy requires that the assay be performed under conditions where receptor reserve and/or stimulus-response coupling efficiency is sufficiently low, such that the maximum effect of the orthosteric agonist in the absence of modulator is below the maximum possible effect attainable in the assay. Under these conditions, modulation of agonist efficacy will then manifest as either a reduction or an increase in the maximum observed response. In contrast, over-expressed or very efficiently-coupled receptor-transducer systems usually result in high degrees of signal amplification such that most agonists utilized behave as full agonists, i.e., yield the maximum possible cellular/tissue response. When the cellular assay system imposes such a ceiling, allosteric enhancement of agonist efficacy would only manifest as an increase in agonist potency, and may be misinterpreted as an allosteric effect on affinity only. Similarly, allosteric inhibition of agonist efficacy in highly amplified signaling assays can result in progressive reductions in potency with no effect on agonist maximum response over the modulator concentration ranges examined. Although effects on agonist maximum response (with/without changes in agonist potency) can be used to infer allosteric modulation of efficacy, an important caveat to the interpretation of functional assays is that the *lack* of such an effect (with/without effects on agonist potency) *cannot* be used as evidence to rule this out, unless it is known that the system under investigation lacks receptor reserve.

### iii) Assessment of Orthosteric Ligand Binding Kinetics

Since the affinity of any ligand for its receptor is determined by the ratio of its association to dissociation rate constants, allosteric interactions that follow the simple ATCM can be detected by comparing the association and/or dissociation rates of a radiolabeled orthosteric ligand in the absence and presence of putative allosteric modulator. Unfortunately, the routine measurement of effects on association kinetics is problematic, because competitive orthosteric ligands will alter the “apparent” association rate simply by delaying the time taken for the radiolabeled probe to reach equilibrium. In contrast, the only way that the dissociation rate of a pre-equilibrated radioligand-receptor complex can be modified is if the test ligand binds to another site on this complex to change receptor conformation prior to the radioligand dissociating.

Radioligand dissociation kinetic assays thus represent a most useful means for detecting and validating an allosteric mode of action. Moreover, under certain conditions these assays can also be used to quantify the allosteric effect in terms of the parameters of the ATCM [52, 60]. Another advantage of these assays is that they have the potential in some cases to detect modulators with neutral binding cooperativity (α = 1) at equilibrium. Neutral cooperativity can arise as a consequence of either a lack of effect on orthosteric ligand association or dissociation rates or**due to the modulator altering *both* properties to the *same* extent. If the latter mechanism is operative, then a dissociation kinetic assay will detect allosteric modulation even when an equilibrium assay will not [51]. However, dissociation kinetic assays are not the be-all and end-all for detecting allosteric modulator effects – there are a number of situations where their utility is limited. The first is when the conformational change induced by the allosteric ligand manifests predominantly on orthosteric ligand association, and not dissociation; without an appropriately designed association kinetic assay, such a modulator would not be detected [62]. The second situation is for interactions characterized by very high negative cooperativity; under this condition, the affinity of the modulator for the radioligand-occupied receptor may be so low such that it cannot bind to perturb dissociation kinetics unless impractically high concentrations of modulator are utilized. A third situation where the dissociation kinetic assay can fail is when the conformational change mediated by the modulator is manifested predominantly on effector coupling domains (i.e. efficacy modulation) and not on the orthosteric binding pocket.

The ability of certain allosteric ligands to alter dissociation of orthosteric ligands from the receptor also has implications for the design and interpretation of “equilibrium” binding studies. The time taken to reach equilibrium is limited by the rate of slowest dissociating ligand [78], thus at very high concentrations of an allosteric modulator that retards orthosteric ligand dissociation, equilibrium may not actually be achieved over the time course of the assay. As a consequence, equilibrium binding experiments may yield complex modulator/radioligand interaction curves that appear inconsistent with the ATCM [3, 60, 84]. In the case of allosteric enhancers, this kinetic artifact can result in a bell-shaped binding curve; for allosteric inhibitors, this can result in a biphasic inhibition curve [ 3].

## PROTOTYPICAL ALLOSTERIC MODULATORS OF THE mAChRs

**Fig. (5):**
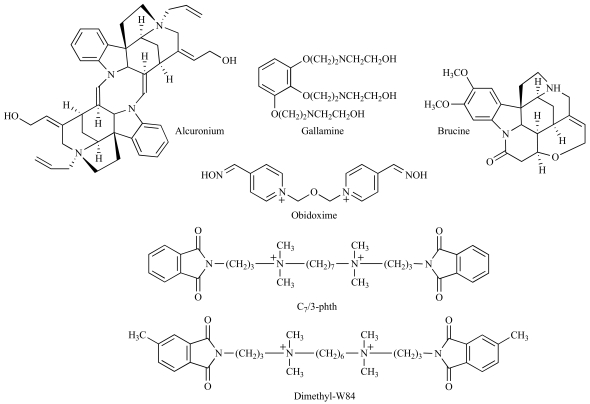
Prototypical “common-allosteric site” mAChR modulators.

Arguably, the most comprehensively studied allosteric modulators of the mAChRs are represented by neuromuscular-blocking agents, such as gallamine and alcuronium, and a series of alkane-bis-onium compounds related to hexamethonium and exemplified by ligands such as W84 and its heptamethylene congener, C_7_/3-phth (Fig. **5**). Collectively, studies with these ligands have resulted in extensive evidence for at least one allosteric site on all five mAChRs that is likely utilized by all these compounds, albeit with significantly different affinities [14, 28]. This will be referred to herein as the “common” allosteric site.

The earliest evidence for allosteric modulation of the mAChRs, and indeed of any GPCR, was obtained in isolated tissue bioassays, specifically, investigations of the effects of alkane-bis-onium modulators and, subsequently, gallamine, at native guinea pig atrial M_2_ mAChRs [19, 70]. The key finding from these early functional assays was that the antagonism by the modulators of orthosteric agonist responses approached a limit at the highest modulator concentrations, resulting in curvilinear Schild regressions. Importantly, with the subsequent widespread adoption of radioligand binding assays, the allosteric properties of these compounds were validated and further studied, confirming that their behavior is generally consistent with the predictions of the simple ATCM. A seminal study of the effects of gallamine on M_2_ mAChRs by Stockton *et al*. [ 94] identified characteristics that have come to be associated with many mAChR modulators, including incomplete inhibition of specific [^3^H]NMS binding at high modulator concentrations and a retardation of the dissociation kinetics of [^3^H]NMS. Subsequent functional and radioligand binding studies have been extensively used to demonstrate the probe-dependence of the allosteric effect, as well as the fact that most of these prototypical common-site modulators have highest affinity for the M_2_ mAChR and lowest affinity for the M_5_ mAChR [ 11,  12,  15,  22, 25,  39,  55, 65,  71,  72].

Another significant finding in the study of mAChR allosterism was the identification of alcuronium as the first allosteric enhancer of the binding of an orthosteric mAChR ligand [84, 101]. This modulator acts at the same site as that recognized by gallamine and the alkane-bis-onium modulators [56, 85], and has proven a very useful tool in demonstrating the striking nature of cooperativity; at the M_2_ and M_4_ subtypes, alcuronium enhances [^3^H]NMS binding, whereas at the M_1_, M_3_ and M_5_ subtypes, it inhibits it [43]. When tested against different orthosteric antagonists and agonists, varying degrees of cooperativity are observed (mostly negative) [ 43,  45,  111]. The alkaloid structure of alcuronium has also prompted investigations into related compounds, leading to the identification of strychnine, vincamine, eburnamonine, and brucine and its analogs as allosteric mAChR modulators [ 59, 86]. Importantly, studies on this series of alkaloids also resulted in the first identification of allosteric enhancers of agonist binding at the mAChRs [ 5,  45,  61]. Obviously, the most important agonist with respect to allosteric modulation is the endogenous neurotransmitter, ACh, and proof-of-concept studies have revealed how positive, neutral and negative cooperativity with this agonist is possible, depending on the modulator and the mAChR subtype [ 5,  45,  61]. Most recently, the identification of thiochrome as a selective allosteric enhancer of ACh at M_4_ mAChRs has added a new dimension to these studies, because the modulator binds with similar affinity at all mAChRs and achieves its selective effect purely from the positive cooperativity between itself and ACh at the M_4_ mAChR [ 64].

Given that mAChR allosteric modulators can display significant degrees of structural diversity, it may be asked whether all these compounds do, indeed, bind to a common allosteric site, or whether they utilize different allosteric sites. The most important pharmacological validation of the common-site hypothesis has been derived from interaction studies between different types of modulators. In particular, the identification of obidoxime (Fig. **5**) and *d*-tubocurarine as allosteric mAChR modulators that bound with reasonable affinity but exerted only a weak effect on radioligand dissociation kinetics [26, 105] meant that they could be used in combination with more efficacious modulators to antagonize the actions of the latter, as would be expected from competition for a common binding site [26, 96, 106].

The most extensive SAR studies focusing on mAChR allosteric modulators has thus led to the following two general categories: neuromuscular blockers and bis-onium modulators, and mono-quaternaries and tertiary amines related to alkaloids; excellent reviews on the SAR of these ligands have been published recently [6, 77]. Other researchers in the field have also used selected members of these prototypical modulator families to design novel pharmacological tools with which to better probe the relationship between the common allosteric site and the orthosteric site on mAChRs. One important approach has been the development of [^3^H]dimethyl-W84 (Fig. **5**), the first radiolabeled allosteric modulator of the M_2_ mAChR [ 97]. This compound may allow for a more direct screening of putative common-site modulators *via* simple competition binding assays [ 98, 99], but has also been used the validate the ATCM as an appropriate mechanistic descriptor of the interaction between the orthosteric site and prototypical common-site modulators [ 98]. Another recent approach is the development of “hybrid” ligands composed of an orthosteric moiety and an allosteric moiety separated by an appropriate covalent linker, which can, theoretically, bind both the orthosteric and allosteric sites. The idea behind this approach is to utilize the allosteric site to achieve selectivity, while still targeting the orthosteric site for the purpose of receptor activation or antagonism [21, 36]. Although the interpretation of the mode of action of these bivalent ligands is likely to be more complex than that predicted by the simple ATCM [ 75], the use of such ligands highlights but one of the many avenues available for selective mAChR targeting *via* exploiting the pharmacology of the prototypical allosteric ligands.

## “ATYPICAL” MODULATORS OF THE mAChRs

**Fig. (6):**
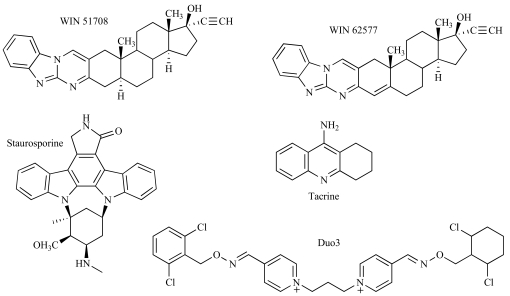
Representative “second-site” and “atypical” mAChR modulators.

In addition to the well-studied common mAChR allosteric site, a second site was more recently defined pharmacologically by Lazareno, Birdsall and colleagues [62, 63]. A number of indolocarbazole derivatives of staurosporine (Fig. **6**), exemplified by the compound, KT5720, were found to show positive, negative and neutral cooperativity with ACh depending on the mAChR subtype, yet did not appear to interact with the prototypical common-site ligands, gallamine and brucine [62]. The novel compounds differ from those reported to act at the common site, in that they generally do not possess a positively charged nitrogen, tend to show highest affinity for the M_1_ rather than M_2_ mAChR, and have little or no effect on [^3^H]NMS dissociation rate. Similarly, analogs of the commercially available neurokinin receptor antagonists, WIN 62,577 and WIN 51,708 (Fig. **6**), as well as the parent compounds themselves, were found to interact with gallamine and strychnine in a non-competitive manner, whilst competing with staurosporine and KT5720 [63]. The WIN compounds also had little or no effect on [^3^H]NMS dissociation, with the exception of the derivative, PG987, which actually accelerated [^3^H]NMS dissociation. A more recent study, focusing predominantly on the M_4_ mAChR, found evidence for a negatively cooperative interaction between WIN 62,577 and each of C_7_/3-phth, alcuronium or brucine when the orthosteric site of the receptor was unoccupied [ 59]. Taken together, these findings indicate that a complex network of cross-interactions is attainable at the mAChRs. It is possible that multiple allosteric sites are also present on other GPCRs.

In addition to the “second-site” modulators described above, there are also a number of other allosteric ligands of the mAChRs that are classed as “atypical” because they exhibit pharmacological behaviors not consistent with the simple ATCM. These compounds include tacrine, the bispyridinium 4,4’-bis-[(2,6-dichloro-benzyloxy-imino)-methyl]-1,1’propane-1,3-diyl-bis-pyridinium dibromide (Duo 3) and a group of pentacyclic carbazolones [35, 81, 96]. Tacrine (Fig. **6**) is a well known anti-cholinesterase that has been reported to inhibit both the equilibrium binding and the dissociation kinetics of [^3^H]NMS with slope factors significantly greater than 1 [ 31,  50,  81, 82,  99]. This behavior is consistent with the expectations of positive homotropic cooperativity, i.e. the binding of one tacrine molecule promotes the binding of another [82]. However, since this behavior is retained in dissociation kinetic assays, where the orthosteric site is occupied by radioligand, the two interacting tacrine molecules must be utilizing two different allosteric sites, perhaps across a mAChR dimer. Alternatively, tacrine is small enough such that two molecules can conceivably bind within the “common” allosteric site. There are two lines of evidence to support the latter conclusion. First, tacrine appears to interact with the common-site modulators obidoxime [ 26] and [^3^H]dimethyl-W84 [ 99]. Second, when two molecules of tacrine are covalently attached to one another to form a dimeric molecule, the affinity of this dimer for the M_2_ mAChR was significantly increased, yet its interaction with [^3^H]NMS no longer showed slope factors greater than 1 [100].

The bispyridinium compound Duo3 (Fig. **6**) is another allosteric mAChR modulator [89] that displays slope factors greater than 1 with respect to inhibition of both [^3^H]NMS and [^3^H]dimethyl-W84, as well as a non-competitive interaction with obidoxime [96, 99]. It has been suggested that Duo3 displays positive homotropic cooperativity, however, unlike tacrine, Duo3 is a large molecule and unlikely to be binding in multiple equivalents within a single, common allosteric site [100]. It is possible that Duo3 represents an allosteric modulator that may exert its effects across receptor dimers, although this remains to be determined.

## ALLOSTERIC EFFECTS ON mAChR SIGNALING AND OTHER BEHAVIORS

As outlined previously, the binding of an allosteric modulator induces a unique receptor conformation that has the potential to not only effect orthosteric ligand affinity, but also efficacy and other receptor behaviors; the abolition by alcuronium of pilocarpine’s efficacy [ 112; see also Fig. (**4**)] is one such example. In addition, certain allosteric ligands may promote or inhibit receptor activation even in the absence of agonist. Indeed, W84 has been shown to be an inverse agonist with respect to [^35^S]GTPγS binding in atrial membranes [ 40]. Alcuronium (at the M_2_ mAChR) and strychnine (at M_1_ and M_2_ subtypes) have both also been identified as inverse agonists with respect to [^35^S]GTPγS binding in recombinant expression systems [ 60,  112]. These findings are generally in accord with the expectation that if a modulator induces a receptor conformation that is negatively cooperative with respect to agonist binding, then the conformation may also predispose the receptor towards a reduced probability of adopting an active state. However, a study by Jakubik *et al*. (1996) [ 44] has found that alcuronium, gallamine, and strychnine were partial (*positive*) agonists at the M_2_, M_4_ and M_1_ mAChR subtypes [ 44]. These findings have not been reported elsewhere, and may reflect particular requirements with respect to receptor-G protein stoichiometry and the use of recombinant expression or artificial reconstitution systems [ 46].

In recent years, there has been an increase in the number of reports identifying putative allosteric agonists of GPCRs. With respect to the mAChRs, McN-A-343 (4-(*m*-Chlorophenylcarbamoyloxy)-2-butynyltrimethylammonium chloride; Fig. (**7**)), probably the first mAChR agonist known to display functional selectivity [87], was actually found to interact allosterically with [^3^H]NMS in an equilibrium radioligand binding assay on rat atrial M_2_ mAChRs over twenty years ago [ 4]. An allosteric mode of interaction with pirenzepine had also been suggested [10], and the agonist was later shown to slow the dissociation kinetics of [^3^H]NMS at cardiac M_2_ mAChRs [ 106]. However, this latter effect was not competitive with d-tubocurarine, and it was suggested that McN-A-343 may in fact bind in two orientations, one to the orthosteric site, and another to an allosteric site (Waelbroeck, 1994). When investigated in functional assays [ 13], the interaction between carbachol and McN-A-343 appeared consistent with simple competition, suggesting that McN-A-343 does indeed recognize the orthosteric site, or else displays very high negative cooperativity against ligands such as carbachol. The ultimate delineation of the mode of action of McN-A-343 as both an agonist and an allosteric modulator is likely to provide novel insights into mAChR activation mechanisms.

**Fig. (7):**
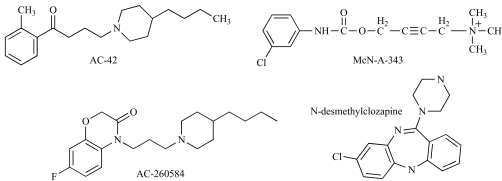
Putative allosteric mAChR agonists.

A number of other agents have more recently been identified as potential mAChR allosteric agonists (Fig. **7**); AC-42 (4-*n*-Butyl-1-[4-(2-methylphenyl)-4-oxo-1-butyl]-piperidine), its analogue AC-260584 (4-[3-(4-butylpiperidin-1-yl)-propyl]-7-fluoro-4*H*-benzo[1, 4]oxasin-3-one and *N*-desmethylclozapine, the major metabolite of the antipscyhotic clozapine. AC-42 displays unprecedented functional selectivity for the M_1_ mAChR relative to all other subtypes, even though it appears to bind with similar affinity for all subtypes. This led to the suggestion that it recognized an “ectopic” site different to that utilized by classic orthosteric ligands [ 93]. A subsequent study by Langmead *et al*. [ 54] provided conclusive evidence for an allosteric mode of action of AC-42. Specifically, the compound was shown to retard the dissociation of [^3^H]NMS from M_1_ mAChRs and, in cell-based functional assays, the antagonism of AC-42-mediated Ca^++^ mobilization at M_1_ mAChRs by atropine was characterized by curvilinear Schild regressions, again consistent with an allosteric mode of interaction [ 54]. Most recently, AC-260584, a more potent AC-42 analogue, was also shown to act allosterically at the M_1_ mAChR [ 92], thus highlighting that a clear SAR is likely to exist that defines allosteric M_1_ mAChR agonism.

Like AC-42, *N*-desmethylclozapine is a functionally-selective M_1_ mAChR agonist that has been suggested to act allosterically. The major lines of evidence for such a mechanism, however, are mainly indirect and based on mutagenesis studies that show differential effects of classic orthosteric site mutations in the M_1_ mAChR on orthosteric ligands such as carbachol, on the one hand, and functionally selective agonists like AC-42 and *N*-desmethylclozapine, on the other [92, 95].

In addition to acute effects on classic signaling pathways, it is now acknowledged that GPCR ligands can affect a far wider range of receptor behaviors that may have a significant impact on the desired therapeutic endpoint. Thus, the pharmacology of a GPCR ligand to impact phenomena such as receptor desensitization, phosphorylation and internalization may not mirror its effects in acute signaling assays [49]. It is of note, therefore, that a recent study found that prolonged exposure of CHO cells stably expressing the human M_2_ mAChR to the allosteric modulators gallamine, alcuronium or C_7_/3-phth, resulted in a significant up-regulation of M_2_ mAChR expression, likely due to an alteration of receptor internalization [ 74].

## MUTATIONAL STUDIES OF THE ALLOSTERIC SITE(S)

There have been two general approaches utilized to map allosteric binding sites on the mAChRs. The most widespread approach has been to use receptor chimeras or site-directed mutagenesis of selected amino acids of one mAChR subtype into their (non-conserved) counterparts of another subtype. The other approach has been to focus on conserved amino acids across mAChR subtypes in order to define residues likely to be critical to the “common” allosteric site at all five subtypes. To date, there have been no reported studies that have aimed to map the location of the “second” allosteric site that is utilized by staurosporine and related compounds.

Since most prototypical (common-site) modulators show highest affinity for the M_2_ mAChR, the bulk of structural studies of mAChR allosteric sites have focused on this subtype, in particular exploiting differences between the M_2_ mAChR and the M_5_ mAChR, since the latter generally displays lowest affinity for many prototypical modulators. Overall, such studies have identified roles for the second and third extracellular loops as well as transmembrane (TM) domain 7 for conferring affinity and selectivity to a diverse range of modulators [8, 27, 29, 37, 42, 47, 52, 67, 100, 104], including gallamine, alkane-bis-onium compounds, alcuronium and d-tubocurarine derivatives. For instance, early site-directed mutagenesis studies revealed the ^172^EDGE^175^ sequence, specific to the second extracellular loop of the M_2_ mAChR, to be required for gallamine selectivity [67]; E^172^ and E^175^ have been highlighted as particularly important, since substitution of these amino acids to their M_1_ counterparts (L and G respectively) resulted in decreased affinity for gallamine and W84 [ 42]. A tyrosine in position 177, also in the second extracellular loop, plays a key role in contributing to the M_2_ versus M_5_ selectivity of WDuo3 [ 100] and binding affinity for diallylcarcurine V and alkane-bis-onium compounds [ 42,  104]. N^419^, at the junction between the third extracellular loop and TM7, plays a role in M_2_ versus M_5_ selectivity of gallamine and W84, although a more dominant residue appears to be the nearby (TM7) T^423^ [ 8,  29,  37,  42,  104]. In terms of conserved residues, a tryptophan in TM7 (W^422^ in the M_2_ mAChR) appears to be the dominant amino acid influencing common-site modulators [ 73,  83].

Similar studies have focused on the differences in modulator activities between the M_3_ and M_2_ mAChRs. Thus, introduction of an asparagine at position 423 of the M_3_ mAChR (corresponding to N^419^ of M_2 _mAChR) resulted in an increase of gallamine’s affinity [ 52]) consistent with the important role that this particular amino acid can play in this position. In addition, N^419^, V^421^ and T^423^ of the M_2_ mAChR were found to be important in the manifestation of positive cooperativity of strychnine-like modulators [ 47]. Collectively, these mutagenesis studies, together with recent homology modeling based on the crystal structure of inactive state bovine rhodopsin [ 58,  83,  104], have resulted in the consensus**view that the common allosteric binding site for the majority of prototypical allosteric M_2 _mAChR modulators is located at the opening of the orthosteric binding pocket, the latter which is buried further within the TM bundle. Fig. (**8**) illustrates the possible relationship between key residues of the orthosteric and allosteric pocket on the M_2_ mAChR, based on homology to bovine rhodopsin.

In contrast to the prototypical modulators, the binding of putative allosteric agonists is believed to be *via* mAChR epitopes distinct from both the orthosteric and common allosteric sites [ 92,  93,  95], although it should be noted that it is far more difficult to interpret the results of mutagenesis studies on agonists because the mutations can affect not only binding affinity, but efficacy as well. Initial studies aimed at investigating the high degree of functional selectivity of AC-42 for the M_1_ mAChR utilized M_1_/M_5_ chimeras, and suggested roles for the N-terminus/TM1 and third extracellular loop/TM7 in AC-42 agonism [ 93]. Additionally, mutagenesis of Y^381^, a key orthosteric site residue in TM6, to Ala of the M_1_ mAChR led to a dramatic reduction in the affinity and potency of carbachol, but had no effect on AC-42 [ 93]. Interestingly, this same mutation actually led to an increase in the agonistic activity of *N*-desmethylclozapine [ 95], clearly indicating that the latter agonist utilizes a different mode of attachment to classic orthosteric ligands, such as carbachol. A more recent study investigating mutations in TM3 known to contribute to the orthosteric site, and which dramatically reduce the efficacy and/or potency of carbachol, found varied effects on the AC-42, AC-260584 and *N*-desmethylclozapine [ 92]. Specifically, a W^101^A substitution increased AC-42 and AC260584 potency and efficacy but had no effect on *N*-desmethyl-clozapine. Mutation of Y^106^A increased the efficacy of *N*-desmethyl-clozapine, whilst S^109^A increased AC-42, AC-260584 and *N*-desmethylclozapine potency [92].

## CONCLUSION

Allosteric modulation of GPCRs represents an exciting and growing field of research, both with respect to drug discovery and a better understanding of GPCR structure and function. The mAChRs remain one of the key model systems for investigating this phenomenon at Family A GPCRs. Not only are there now a good number of structurally diverse allosteric modulators identified for this receptor family, but the receptors themselves remain important therapeutic candidates that have yet to be optimally targeted, thus ensuring an impetus for additional exploration of allosteric ligand chemical space. As with many nascent fields, however, significant challenges remain. The prevalence and relevance of allosteric agonists of the mAChRs, for example, has not been fully gauged as yet. Mutagenesis and molecular modeling studies aimed at mapping putative allosteric sites, with a view towards relating structure to function and identifying novel ligands, still have much ground to cover. Nonetheless, the potential rewards are significant and, as such, the study of mAChR allosterism remains one that is likely to deliver significant pharmacological dividends.

## Figures and Tables

**Fig. (1):**
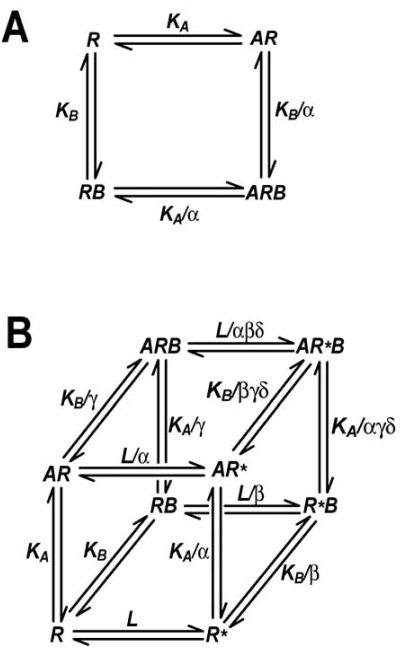
Allosteric GPCR models. **A)** The simple allosteric ternary complex model (ATCM), which describes the interaction between an orthosteric ligand, A, and allosteric modulator, B, in terms of their equilibrium dissociation constants (K_A_, K_B_) and the cooperativity factor, α, which describes the magnitude and direction of the allosteric effect on ligand binding affinity. **B)** The allosteric two state model (ATSM), which describes allosteric modulator effects on affinity, efficacy and the distribution of the receptor between active (R*) and inactive (R) states, in terms of distinct conformations selected by ligands according to their cooperativity factors for the different states.

**Fig. (4):**
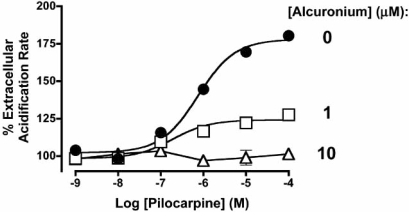
Allosteric modulation of orthosteric agonist efficacy. Interaction between alcuronium and pilocarpine at human M_2_ mAChRs stably expressed in CHO cells. Receptor activation was quantified as a change in the extracellular whole cell acidification rate with a Cytosensor microphysiometer.

**Fig. (8):**
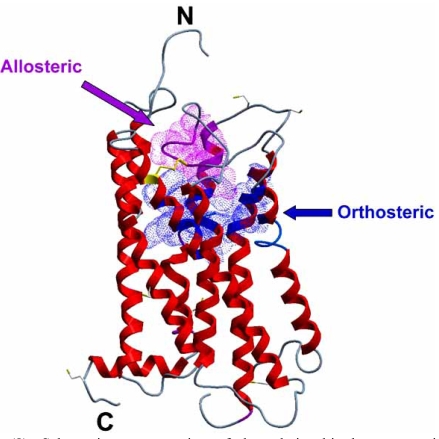
Schematic representation of the relationship between residues comprising the orthosteric and “common” allosteric site on the M2 mAChR, using a homology model based on the crystal structure of inactive state bovine rhodopsin. Regions highlighted in blue incorporate the following orthosteric-site residues: W^99^, D^103^, S^107^, Y^110^, W^155^, T^187^, T^190^, W^400^, Y^403^, N^404^, Y^426^, Y^430^. Regions highlighted in purple incorporate the following allosteric site residues: ^172^EDGE^175^, Y^177^, N^419^, N^422^, N^423^. The residues in yellow represent a cysteine pair, and corresponding disulphide bond between the second extracellular loop and top of TM3, that are highly conserved in over 90% of GPCRs.
